# Clinical outcomes of intravitreal treatment for ocular toxoplasmosis: systematic review and meta-analysis

**DOI:** 10.1590/0037-8682-0552-2022

**Published:** 2023-05-22

**Authors:** Lutiana Amaral de Melo, Mayara Rodrigues Brandão de Paiva, Gabriella Maria Fernandes-Cunha, Armando Silva-Cunha, Marcos Paulo Gomes Mol, Sílvia Ligorio Fialho

**Affiliations:** 1 Fundação Ezequiel Dias, Diretoria de Pesquisa e Desenvolvimento, Belo Horizonte, MG, Brasil.; 2 Byers Eye Institute at Stanford University School of Medicine, Ophthalmology, Palo Alto, CA, United States.; 3 Universidade Federal de Minas Gerais, Faculdade de Farmácia, Belo Horizonte, MG, Brasil.

**Keywords:** Intravitreal, Toxoplasmosis, Ocular, Meta-analysis, Systematic review

## Abstract

**Background:**

Ocular toxoplasmosis is the leading cause of infectious posterior uveitis worldwide, accounting for 30-50% of all cases in immunocompetent patients. Conventional treatment is associated with adverse effects and does not prevent recurrence. Intravitreal drug administration can improve disease outcomes and reduce side effects. Herein, we conducted a systematic review and meta-analysis on the efficacy of intravitreal injections for treating ocular toxoplasmosis.

**Methods:**

The systematic search was conducted using PubMed, SciELO, and Google Scholar with the descriptors “ocular toxoplasmosis” AND “intravitreal”. We analyzed studies that met the inclusion criteria, i.e., experimental cases in patients treated intravitreally for ocular toxoplasmosis. Considering the systematic review, we focused on the number of intravitreal injections, the therapeutic drug class, and the presence of preexisting conditions. To assess the efficacy of intravitreal injections, a meta-analysis was performed using visual acuity, side effects, disease recurrence, and inflammatory responses as variables.

**Results:**

Intravitreal injection-induced side effects were rarely observed (0.49% [0.00, 1.51%] ). The use of antiparasitic and anti-inflammatory drugs afforded improved visual acuity (99.81% [98.60, 100.00%]) and marked effectiveness in treating ocular toxoplasmosis.

**Conclusions:**

Intravitreal injections may facilitate the successful treatment of ocular toxoplasmosis. However, clinicians should carefully evaluate the presence of preexisting conditions for ocular toxoplasmosis or previous diseases, as these can impact the decision to administer intravitreal injections.

## INTRODUCTION

Ocular toxoplasmosis, a disease caused by the parasite *Toxoplasma gondii*, is considered the primary cause of infectious posterior uveitis worldwide and is responsible for 30-50% of all cases of posterior uveitis in immunocompetent patients^1^ ^,^ ^2^. Infections can be congenital or acquired, and studies have shown that most cases can be attributed to infection after birth^3^ ^,^ ^4^. Despite the high global prevalence of ocular toxoplasmosis and its burden on patients, this disease remains neglected as a common health problem, owing to the complexity of its pathophysiology, eye immunology, and recurrence of infection^5^. 

In acute infection, tachyzoites of the parasite penetrate the eye tissue, causing inflammation, necrosis, scarring, atrophy of the retina and choroid (retinochoroiditis), and inflammation of the optic nerve head (papillitis) and uvea (uveitis)^6^. If not properly treated, this infection can lead to severe visual impairment, depending on the immunological state of the individual. In immunocompetent patients, the infection usually heals within six to eight weeks. To escape the host immune system, the parasite can become dormant, transform into bradyzoites, and form cysts, leading to lifelong infection. In chronic ocular toxoplasmosis, parasitic cysts can be found in the retina, ganglion, and Muller cells^7^. Typically, the disease recurrence rate after the first episode is 5 years^8^, although 50% of recurrences occur within 3 years of the first episode^7^. 

In the last few decades, research has focused on discovering new drugs exerting anti-toxoplasma activity and improving treatment regimens with existing drugs. The common treatment for ocular toxoplasmosis is an oral combination of (1) pyrimethamine and sulfadiazine, (2) pyrimethamine with clindamycin, azithromycin, atovaquone, or (3) trimethoprim with sulfamethoxazole^8^. However, this combination therapy is frequently associated with severe adverse effects and toxicity. Moreover, these treatment regimens fail to prevent disease recurrence. Local treatment of eye infections using intravitreal drug administration is gaining attention owing to improved patient outcomes and a marked reduction in side effects^9^ ^,^ ^10^. Over the past 10 years, we have investigated the benefits of locally delivered drugs to treat ocular toxoplasmosis. Recently, we have shown that the slow-release clindamycin implants were safe for intravitreal use and may have contributed to the long-term control of toxoplasmosis chorioretinitis^11^. The present meta-analysis and systematic review summarizes the currently employed local treatments for ocular toxoplasmosis and provides a comprehensive discussion of clinical outcomes and control. 

## METHODS

This study was conducted in accordance with the MOOSE (Meta-Analyses of Observational Studies in Epidemiology) guidelines and PRISMA (Preferred Reporting Items for Systematic Reviews and Meta-Analyses) statement^12^.

### Search methods

The search included the main electronic databases: PubMed, SciELO, and Google Scholar. Publications available up to February 2022 were assessed, and respective citations were also followed up. Although English was the preferred language for published papers, Spanish was also considered. The following descriptors were used: “ocular toxoplasmosis" AND intravitreal.

### Selection criteria

Articles reporting experimental cases in patients who were intravitreally administered drugs for treating ocular toxoplasmosis were included, with no restrictions on publication date, age, race, sex of patients, or region. Articles were initially screened by title, followed by abstracts, and finally, the full text was evaluated. The statistical analysis adopted 95% confidence intervals (CI) to calculate the prevalence of each outcome. Three independent reviewers rigorously screened titles, abstracts, and full texts. Disagreements were resolved through discussions until a consensus was reached. 

Articles that met any of the following criteria were excluded: 1) route of administration other than intravitreal; 2) *in vitro* and animal studies; 3) reviews, systematic reviews, or meta-analyses; 4) abstracts or studies with no available full text; 5) diseases other than ocular toxoplasmosis; 6) studies published in languages other than English or Spanish; and 7) duplicates.

### Quality assessment

The quality of included articles was evaluated using the adapted guidelines for critically appraising studies on the prevalence or incidence of health problems and the GRADE approach^13^. 

The articles received scores ranging from 0 to 6 points according to the following aspects: study design, sampling method, standard criteria, response rate, CIs, and setting. The scores were assigned to each aspect by consensus of reviewers and varied from very low (0-1), low (2-3), moderate (4-5), to high (6), depending on the quality level of the included article.

### Statistical methods

The systematic review was performed using all papers identified in the literature on the use of intravitreal injections for treating ocular toxoplasmosis. For the meta-analysis, we only used articles that reported more than one patient as a requirement of the prevalence analysis model. Forest plot models were used to calculate the efficacy of the main outcomes.

The main variables evaluated in the present meta-analysis were improved vision, reduced side effects, and inflammation. Heterogeneity between studies was evaluated using the chi-square based on the Q test. The Q statistic was approximately distributed, similar to the x^2^ distribution, with k-1 degrees of freedom. Fixed- and random-effects models were used to determine the absence or presence of heterogeneity between studies. A Funnel-Plot was used to verify publication bias, which was confirmed using linear regression^14^. The main results were presented as forest plots. We used the metafor package in R software version 4.0.3^15^.

Meta-analysis variables were converted to prevalence values to create forest plots. Only fitted models are presented, which were observed for improved vision and side-effect variables. For the analysis, we considered all improvements observed in best-corrected visual acuity or similar tests, as well as side effects described by authors in selected studies (Tables 1 and 2)^9^ ^,^ ^16^ ^-^ ^61^. According to the prevalence analysis model, a forest plot model was proposed for studies reporting more than one patient.

**TABLE 1:** Articles included in the systematic review with more than one patient reported per study.

Author	Pharmacological class	Average of interventions	Treatment time (average days)	Age (years)	Female	Male	Associated conditions	Patients	Improved eyesight	Adverse reactions	Inflammation reduction	Quality assessment
Benevento et al, 2008^16^	anti-VEGF	more than one (2)	30	7, 25	NA	NA	0	2	1	0	NA	5
Martinez-Castillo et al., 2012^17^	antiparasitic and anti-inflammatory	more than one (1)	19	30, 34	1	1	pregnancy and sulfa intolerance	2	2	NA	2	4
Crosson et al., 2020^18^	antiparasitic	more than one (1)	30	74-77	1	1	diabetes	2	0	NA	2	4
Cordero-Coma et al., 2010^19^	anti-VEGF	more than one (2)	120	19, 44	2	0	0	2	NA	0	NA	4
Choudhury et al, 2015^20^	antiparasitic and anti-inflammatory	more than one (4)	7	12-41	1	3	0	4	4	0	4	5
Lin et al., 2011^21^	anti-VEGF	1	1	24-26	1	1	0	2	2	0	2	5
Cortés et al., 2019^22^	antiparasitic and anti-inflammatory	1	90	44, 67	0	2	0	2	0	1	2	5
Petrou et al., 2013^23^	anti-VEGF	more than one (2)	7	21, 52	1	1	0	2	1	0	NA	5
Soheilian et al, 2011^24^	antiparasitic and anti-inflammatory	more than one (16)	24	24.5±6.0	18	16	0	34	34	0	34	6
Ocampo Dominguez, 2015^25^	antiparasitic	1	NA	NA	6	16	0	22	18	5	22	6
Zamora et al., 2015^26^	antiparasitic	more than one (4)	270	34.0±13.5	10	6	0	16	14	0	2	6
Aggio et al., 2006^27^	anti-inflammatory	1	1	30, 74 74	1	1	diabetes	2	2	0	2	5
Souza et al, 2017^28^	antiparasitic	more than one (5)	30	21-76	9	4	0	13	13	0	13	6
Baharivand et al, 2013^29^	antiparasitic and anti-inflammatory	1	NA	25,6±4,0	17	15	0	32	32	0	32	6
Sobrin et al., 2007^30^	antiparasitic	1	1	23-52	0	1	0	6	5	1	6	5
Verma et al., 2020^10^	antiparasitic	more than one (4)	7	12-57	2	2	0	4	3	0	4	5
Lasave et al., 2010^31^	antiparasitic and anti-inflammatory	more than one (12)	50	31,9 ±11,3	9	3	pregnancy	12	12	0	12	6
Bor’i et al., 2018^32^	antiparasitic and anti-inflammatory	NA	60	35.5±4.1	9	6	0	30	30	0	30	6
Kianersi et al., 2015^33^	anti-VEGF	1	1	16-32	2	2	0	4	4	0	NA	5
Ben Yahia et al., 2008^34^	anti-VEGF	1	1	25, 26	2	0	0	2	2	0	NA	5
Korol et al., 2017^35^	anti-VEGF	more than one (10)	28	16-56	11	3	NA	14	14	3	NA	6
Hegde et al., 2015^36^	anti-VEGF	more than one (2)	30	15, 51	2	1	0	3	3	0	3	5
Kianersi et al., 2015^37^	anti-VEGF	1	1	16-37	3	2	0	5	5	0	NA	6

NA: not applicable; anti-VEGF: anti-vascular endothelial growth factor.

**TABLE 2:** Articles included in the systematic review with only one patient reported per study.

Author	Pharmacological class	Average of interventions	Treatment time (average days)	Age	Female	Male	Associated conditions	Improved eyesight	Adverse reactions	Inflammation reduction
Jorge et al., 2021^11^	antiparasitic and anti-inflammatory	more than one	120	45	1	0	HIV positive	Yes	No	Yes
Anaya and Castro, 2019^38^	antiparasitic	more than one	NA	33	0	1	-	Yes	Yes	Yes
Santos, 2008^39^	antiparasitic and anti-inflammatory	1	NA	34	1	0	-	Yes	No	Yes
Khandwala et al., 2021^40^	anti-VEGF	1	150	17	1	0	-	Yes	No	Yes
Wong et al., 2009^41^	antiparasitic	more than one	15	25	1	0	-	No	No	Yes
Henao-Martínez et al., 2018^42^	antiparasitic	1	NA	30	1	0	-	NA	No	Yes
Martinez et al., 1998^43^	antiparasitic and anti-inflammatory	more than one	28	17	1	0	pregnancy	Yes	No	Yes
Fonollosa et al., 2016^44^	anti-inflammatory	more than one	NA	55	0	1	HIV positive	Yes	Yes	Yes
Mushtaq et al., 2019^45^	anti-VEGF	more than one	90	22	1	0	-	Yes	No	Yes
Mehta et al., 2018)^46^	antiparasitic	1	NA	33	0	1	-	Yes	No	Yes
Mathur et al., 2014^47^	anti-VEGF	more than one	NA	13	1	0	-	Yes	No	Yes
Perez et al., 2020^48^	antiparasitic and anti-inflammatory	1	42	14	1	0	-	Yes	No	Yes
Martín García et al., 2020^49^	anti-VEGF	more than one	15	12	0	1	-	Yes	No	Yes
Bawdekar et al., 2013^50^	anti-inflammatory	more than one	3	39	0	1	HIV positive	Yes	No	Yes
Shah and Shah, 2011^51^	anti-VEGF	1	1	18	1	0	-	Yes	No	NA
Hosseini et al., 2014^52^	antiparasitic	1	1	23	1	0	-	Yes	No	Yes
Tabuenca del Barrio et al., 2019^9^	antiparasitic	more than one	28	27	1	0	sulfa intolerance	NA	NA	NA
Kim et al., 2002^53^	antiparasitic and anti-inflammatory	1	1	57	1	0	sulfa intolerance	NA	Yes	NA
Abrishami et al., 2021^54^	antiparasitic and anti-inflammatory	1	1	32	0	1	-	Yes	No	Yes
Dutta Majumder et al., 2021^55^	antiparasitic	1	1	35	0	1	-	Yes	No	Yes
Ghassemi et al., 2014^56^	antiparasitic	1	1	4	0	1	retinoblastoma	NA	NA	NA
Rishi et al., 2011^57^	anti-VEGF	1	1	14	2	0	-	Yes	No	NA
Theodoropoulou et al., 2012^58^	antiparasitic	more than one	NA	30	0	1	-	NA	Yes	Yes
Raval Vishal, Rao Srinivas, 2018^59^	antiparasitic	more than one	42	39	0	1	-	No	No	No
Sánchez Vega et al., 2014^60^	antiparasitic	1	1	16	0	1	-	No	Yes	Yes
Hazan et al., 2013^61^	antiparasitic	more than one	4	27	0	1	-	Yes	No	Yes

NA: not applicable; anti-VEGF: anti-vascular endothelial growth factor.

## RESULTS

**FIGURE 1:**
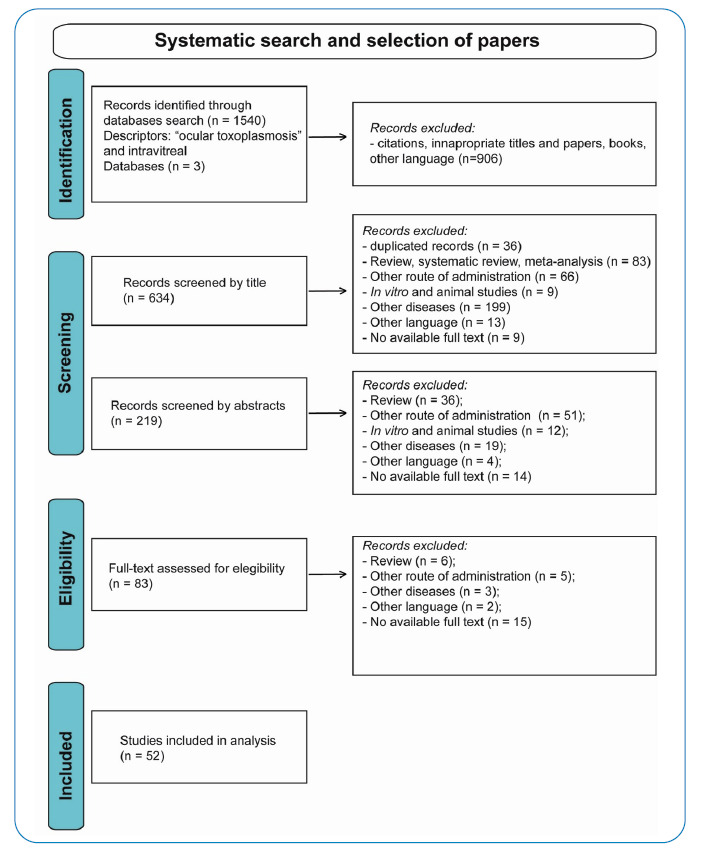
Flow chart of articles selection stages.

The flowchart in Figure 1 illustrates the stages of the systematic search and article selection in the present study. Fifty-two articles reporting the intravitreal treatment of ocular toxoplasmosis are listed in Tables 1 and 2. 

Considering the included studies, a total of 119 females and 97 males with ocular toxoplasmosis underwent intravitreal drug administration (Tables 1 and 2); however, sex did not impact the treatment choice. Regarding the number of intravitreal injections, of the 52 studies included, 24 (46%) performed a single intravitreal injection, and 27 (53.5%) studies performed two or more (up to a maximum of 6) intravitreal injections; this information was unavailable for one study (0.5%) (Tables 1 and 2). Administering multiple intravitreal injections has been shown to cause patient discomfort and increase the risk of severe side effects, such as endophthalmitis, damage to the posterior capsule of the lens, and retinal detachment^29^ ^,^ ^61^. In the present review, we found that the number of injections performed varied from one to six, with an average of two weeks between doses. Retinal detachment was the most frequently reported side effect, followed by cataracts, pain, rash, and hemorrhage. Despite these adverse effects, we could not establish whether the observed complications were related to the number of intravitreal injections administered (Tables 1 and 2). Of the 52 included studies, two employed clindamycin/dexamethasone or dexamethasone implants to reduce the side effects associated with frequent intravitreal injections; in both these studies, patients were HIV positive. Although strategies to treat ocular toxoplasmosis may differ in patients with HIV, intravitreal treatment demonstrated a decrease in posterior uveitis in both studies, along with no disease recurrence reported during a follow-up period of at least 18 months^11^ ^,^ ^44^. 

Another parameter evaluated was the presence of preexisting conditions associated with ocular toxoplasmosis, which could eventually influence the choice of intravitreal therapy. Considering 13 (25%) included articles, patients had diabetes, were pregnant, had an allergy to sulfa, or had positive serology for HIV. One patient presented with retinoblastoma coexisting with ocular toxoplasmosis, whereas another had undergone transplantation (Tables 1 and 2). Unfortunately, most studies failed to report the presence or absence of preexisting conditions associated with ocular toxoplasmosis. Therefore, the influence of this parameter on the treatment choice was not considered. However, it has been previously reported that intravitreal injections could be administered to patients with contraindications for oral therapy, including intolerance, allergy, or severe side effects of the classic oral treatment or if the infection is resistant to the drug regimen^10^ ^,^ ^62^ ^-^ ^64^.

**FIGURE 2:**
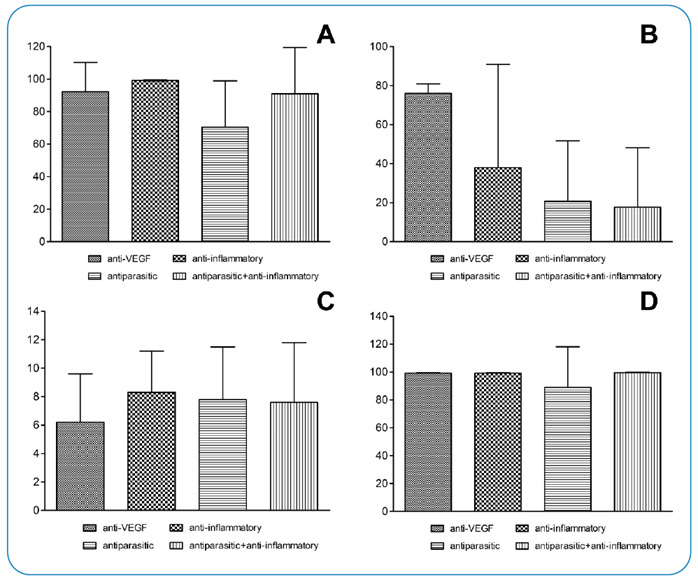
Descriptive statistical data regarding the prevalence (%) of improved eyesight **(A)**, adverse reactions **(B)**, recurrence **(C)**, and inflammation reduction **(D)**, according to therapeutic class. anti-VEGF: anti-vascular endothelial growth factor.

To facilitate data presentation, we grouped the main drugs administered intravitreally into four classes: antiparasitic (19), anti-vascular endothelial growth factor (VEGF) (17), anti-inflammatory in combination with antiparasitic (13), and anti-inflammatory (3) drugs. The values in parentheses indicate the number of included articles per drug class. Among articles evaluated in the classes of antiparasitic drugs alone or combined with anti-inflammatory drugs (32), clindamycin was the most commonly used drug, employed in 91% (29) of articles, followed by sulfamethoxazole and trimethoprim (9%; 3). Considering studies in which patients received anti-inflammatory drug class (30.8%, 16), dexamethasone was the most commonly used drug (87.5%; 14/16), followed by triamcinolone and triamcinolone plus dexamethasone (12.5%; 2/16). Overall, 33% of studies (17) evaluated the use of intravitreal anti-VEGF agents; however, these were not included in our meta-analysis. We believe that anti-VEGF injections can be used to treat the secondary effects of ocular toxoplasmosis. However, anti-VEGF injections are important for controlling disease-related side effects; therefore, these studies were included in the systematic review. In addition, the exclusion criteria did not include the therapeutic classes of drugs. Bevacizumab (8/17) was the most commonly administered anti-VEGF drug, followed by ranibizumab (5/17). One study used aflibercept, and two used bevacizumab or ranibizumab, with the drug not described in one study. Figure 2 presents the impact of the therapeutic class of drugs on visual acuity, side effects, disease recurrence, and inflammatory responses. 

**FIGURE 3:**
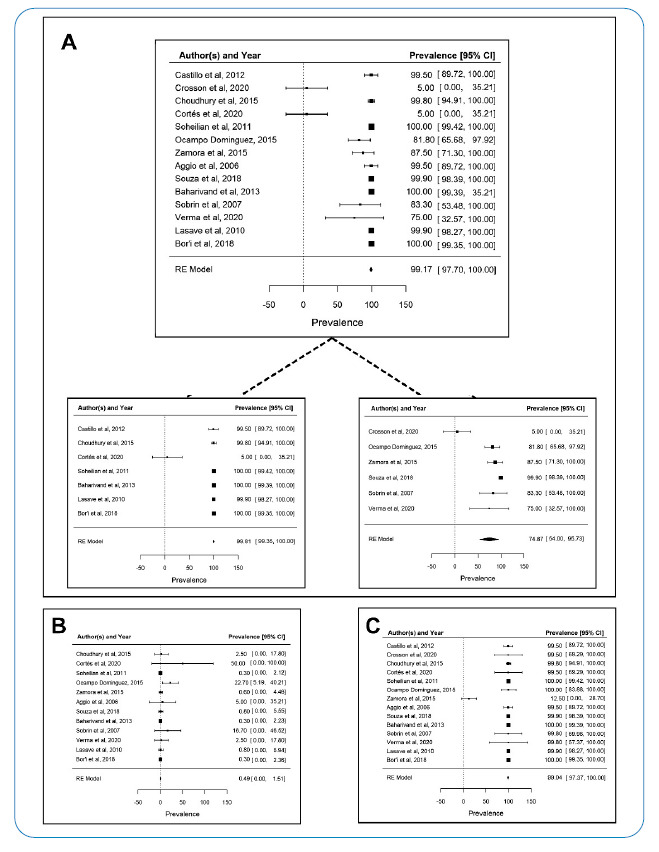
**A:** Forest plot of improvement in visual acuity prevalence (%) for all included papers by meta-analysis (above) and according to therapeutic class, anti-inflammatory associated with antiparasitic (left), and antiparasitic (right); **B:** Forest plot of adverse effects prevalence (%) for all included papers; **C:** Forest plot of reduced ocular inflammation prevalence (%) for all included papers. **CI:** confidence interval.

Only 84% of patients who received an antiparasitic drug alone (74.87% [54.00, 95.73%]) Figure 3 (A - right) had improvements in visual acuity when compared with 90% of the patients who received an antiparasitic drug combined with an anti-inflammatory drug (99.81% [98.60, 100.00%]), as shown in Figure 3 (A - left), and 100% of patients who received an anti-inflammatory drug alone. The forest plot shown in Figure 3 (A - above) was validated (p = 0.0030), with I^2^= 84.83% (high heterogeneity among studies). Egger's test confirmed the absence of publication bias (linear regression, p<0.001).

Interestingly, articles that described the use of antiparasitic drugs alone (19/52) reported 70% effectiveness in treating ocular toxoplasmosis, compared with 90% effectiveness reported by those that used an antiparasitic drug combined with a corticosteroid (13/52). In addition, 10 of the 13 studies revealed that dexamethasone combined with clindamycin effectively treated toxoplasmosis retinochoroiditis. Of the 13 studies, one study exploring trimethoprim/sulfamethoxazole and dexamethasone injected intravitreally reported improved visual acuity. Raval et al. reported that administering intravitreal trimethoprim/sulfamethoxazole alone was insufficient to treat ocular toxoplasmosis^59^. Therefore, we suggest that intravitreal administration of an antiparasitic drug combined with corticosteroids could increase the treatment efficacy for ocular toxoplasmosis^20^ ^,^ ^28^. 

The frequency of side effects related to intravitreal injections was low (0.49% [0.00, 1.51%] ), as summarized in Figure 3B. The forest plot was validated (p = 0.031) with I^2^= 48.17% (low heterogeneity among the studies) (Figure 3B). Egger's test confirmed the absence of publication bias (linear regression, p=0.0264). However, a high number of side effects were noted in articles (35/52) assessing the concomitant administration of systemic therapy and intravitreal injections.

The prevalence of reduced ocular inflammation was 99.04% [97.37, 100.00]%), as shown in Figure 3C, with a p-value of 0.0001 and I^2^= 88.39%. As expected, this therapeutic effect was related to the use of anti-inflammatory drugs to treat infections.

## DISCUSSION

Currently, there is no effective treatment for ocular toxoplasmosis capable of eradicating *T. gondii* infection. More than 50% of patients with ocular toxoplasmosis experience disease recurrence, which can cause progressive vision loss and decrease their quality of life^65^ ^,^ ^66^. Conventional therapy is based on the combination of several systemic drugs that can cause major side effects, thereby reducing patient compliance and leading to decreased treatment efficacy and increased chances of disease recurrence^65^ ^,^ ^66^ ^,^ ^67^. Alternatively, oral sub-doses of trimethoprim-sulfamethoxazole can reduce the recurrence of ocular toxoplasmosis^68^ ^,^ ^69^. However, intravitreal injections can deliver high drug concentrations to the eye, thereby reducing systemic absorption and side effects. Thus, intravitreal injections may afford an alternative therapeutic strategy for ocular toxoplasmosis^11^ ^,^ ^70^ ^,^ ^71^ ^,^ ^72^.

Over the last few years, systematic reviews and meta-analyses have described the clinical effectiveness of anti-toxoplasma therapies based on therapeutic approaches and current treatments^6^ ^,^ ^22^ ^,^ ^67^ ^,^ ^73^ ^-^ ^77^. In a systematic review, Jasper et al. reported treatment outcomes following antiparasitic therapy with or without systemic corticosteroids^6^. A meta-analysis by Zhang et al. evaluated the most effective therapy for ocular toxoplasmosis in immunocompetent patients^65^. Another systematic review and meta-analysis by Feliciano-Alfonso et al. examined the effects and safety of existing drug regimens in treating ocular toxoplasmosis^77^. However, to date, no systematic reviews or meta-analyses have evaluated the effectiveness of intravitreal drug administration for treating ocular toxoplasmosis.

In the present study, we performed a systematic review and meta-analysis discussing the most recent information on the clinical outcomes related to the use of intravitreal injections to treat ocular toxoplasmosis. 

This systematic review focused on the most relevant parameters for selecting intravitreal administration. These parameters included the number of intravitreal injections, the therapeutic class of drugs, and the presence of other preexisting conditions. To assess the efficacy of intravitreal injections, a meta-analysis was performed using the following variables: visual acuity, side effects, disease recurrence, and inflammation response. 

Previous studies have demonstrated that conventional treatment for toxoplasmosis, which includes combination therapy with several drugs, is commonly associated with side effects and toxicity. Patients treated with pyrimethamine and sulfadiazine experience side effects such as hematologic toxicity, rash, and fever, which can lead to the discontinuation of therapy^8^ ^,^ ^78^ ^,^ ^79^. In addition, oral clindamycin can cause vomiting and/or diarrhea^10^. Extended treatment and challenges in eliminating the infection can complicate current treatment strategies, emphasizing the need for new alternatives. Intravitreal injections are considered feasible alternatives for controlling ocular toxoplasmosis. Complications of intravitreal injection include retinal injury, detachment, endophthalmitis, and lens damage, which can be avoided by adopting appropriate procedures for intravitreal administration and sterilization, as described in the available guidelines^10^ ^,^ ^29^ ^,^ ^61^. Furthermore, it has been previously reported that intravitreal therapy may be more convenient and safe for patients^24^ ^,^ ^29^, increasing patient compliance. 

Herein, we found that intravitreal injections may contribute to the successful treatment of ocular toxoplasmosis. In addition, we found that the therapeutic class of drugs influenced visual acuity. Most included studies have failed to report the presence of preexisting conditions for ocular toxoplasmosis or previous diseases. This can be considered a limitation of the present study, given that this information is essential for deciding on the use of intravitreal injection. Another limitation was the inclusion of studies assessing only one patient in the meta-analysis; this reduced the sample size. However, we used methodological steps to validate the results.

## References

[1] Robert-Gangneux F, Dardé ML (2012). Epidemiology of and diagnostic strategies for toxoplasmosis. Clin Microbiol Rev.

[2] Pfaff AW, de-la-Torre A, Rochet E, Brunet J, Sabou M, Sauer A (2014). New clinical and experimental insights into Old World and neotropical ocular toxoplasmosis. Int J Parasitol.

[3] Delair E, Monnet D, Grabar S, Dupouy-Camet J, Yera H, Brézin AP (2008). Respective Roles of Acquired and Congenital Infections in Presumed Ocular Toxoplasmosis. Am J Ophthalmol.

[4] Delair E, Latkany P, Noble AG, Rabiah P, McLeod R, Brézin A (2011). Clinical manifestations of ocular toxoplasmosis. Ocul Immunol Inflamm.

[5] Smith JR, Ashander LM, Arruda SL, Cordeiro CA, Lie S, Rochet E (2021). Pathogenesis of ocular toxoplasmosis. Prog Retin Eye Res.

[6] Jasper S, Vedula SS, John SS, Horo S, Sepah YJ, Nguyen QD (2017). Corticosteroids as adjuvant therapy for ocular toxoplasmosis. Cochrane Database Syst Rev.

[7] Song HB, Jung BK, Kim JH, Lee YH, Choi MH, Kim JH (2018). Investigation of tissue cysts in the retina in a mouse model of ocular toxoplasmosis: distribution and interaction with glial cells. Parasitol Res.

[8] Smith NC, Goulart C, Hayward JA, Kupz A, Miller CM, van Dooren GG (2021). Control of human toxoplasmosis. Int J Parasitol.

[9] Tabuenca Del Barrio L, Mulero HH, Mozo Cuadrado M, Fanlo Mateo P, Compains Silva E (2019). Intravitreal clindamycin as a therapeutic alternative in severe ocular toxoplasmosis. Arch Soc Esp Oftalmol.

[10] Verma L, Thulasidas M, Gupta A (2020). Intravitreal clindamycin as first-line therapy for toxoplasmic retinochoroiditis: A case series. Clin Ophthalmol.

[11] Jorge R, Coelho IN, Silva-Cunha A, Fernandes Cunha GM, Scott IU, Fialho SL (2021). Use of a slow-release intravitreal clindamycin implant for the management of ocular toxoplasmosis. Am J Ophthalmol Case Rep.

[12] Moher D, Liberati A, Tetzlaff J, Altman DG (2009). Preferred Reporting Items for Systematic Reviews and Meta-Analyses: The PRISMA Statement. PLoS Med.

[13] Loney PL, Chambers LW, Bennett K, Roberts JG, Stratford PW (1998). Prevalence of incidence of a health problem. Chronic Dis Can.

[14] Light RJ, Pillemer D (1984). Summing Up: The science of Reviewing Research.

[15] Viechtbauer W (2010). Conducting Meta-Analyses in R with the metafor Package. J Stat Softw.

[16] Benevento JD, Jager RD, Noble AG, Latkany P, Mieler WF, Sautter M (2008). Toxoplasmosis-Associated Neovascular Lesions Treated Successfully with Ranibizumab and Antiparasitic Therapy. Arch Ophthalmol.

[17] Martinez-Castillo S, Gallego-Pinazo R, Francés-Munhoz E, Dolz-Marco R, Vásquez-Polo A, Diaz-Llopis M (2012). Macular toxoplasmosis and intravitreal clindamycin: an altenative to oral treatment. Arch Soc Esp Oftalmol.

[18] Crosson JN, Kuthyar S, Shantha JG, Debiec MR, Laird PW, Hwang CS (2020). Toxoplasmosis chorioretinitis mimicking acute retinal necrosis associated with local corticosteroid. Int J Retin Vitr.

[19] Cordero-Coma M, Pérez E, Calleja S, García Ruiz de Morales JM (2010). Toxoplasmic retinochoroiditis: relapse vs choroidal neovascular membrane. Arch la Soc Española Oftalmol.

[20] Choudhury H, Jindal A, Pathengay A, Bawdekar A, Albini T, Flynn HW (2015). The role of intravitreal trimethoprim/sulfamethoxazole in the treatment of toxoplasma retinochoroiditis. Ophthalmic Surg Lasers Imaging Retin.

[21] Lin C, Chen S-N, Hwang J-F, Hu P-S (2011). Successful Treatment of Toxoplasmosis-Associated Choroidal Neovascular Lesions With Bevacizumab and Antiparasitic Therapy. Retin Cases Brief Rep.

[22] Cortés JA, Roncancio Á, Uribe LG, Cortés-Luna CF, Montoya JG (2019). Approach to ocular toxoplasmosis including pregnant women. Curr Opin Infect Dis.

[23] Petrou P, Georgalas I, Markomichelakis N, Vergados I, Gianakaki E, Rouvas A (2013). Resolution of vitreomacular traction following intravitreal ranibizumab in cases of ocular toxoplasmosis with choroidal neovascularization. Ther Clin Risk Manag.

[24] Soheilian M, Ramezani A, Azimzadeh A, Sadoughi MM, Dehghan MH, Shahghadami R (2011). Randomized trial of intravitreal clindamycin and dexamethasone versus pyrimethamine, sulfadiazine, and prednisolone in treatment of ocular toxoplasmosis. Ophthalmology.

[25] Ocampo Dominguez HH (2015). Manejo de Toxoplasmosis Ocular Severa con Clindamicina y Triamcinolona Intravitreas: Reporte de 22 Casos. Rev la Soc Colomb Oftalmol.

[26] Zamora YF, Arantes T, Reis FA, Garcia CR, Ferreira Saraceno JJ, Belfort R (2015). Local treatment of toxoplasmic retinochoroiditis with intravitreal clindamycin and dexamethasone. Arq Bras Oftalmol.

[27] Aggio FB, Muccioli C, Belfort R (2006). Intravitreal triamcinolone acetonide as an adjunct in the treatment of severe ocular toxoplasmosis.

[28] Souza CE, Nascimento H, Lima A, Muccioli C, Belfort R (2018). Intravitreal Injection of Sulfamethoxazole and Trimethoprim Associated with Dexamethasone as an Alternative Therapy for Ocular Toxoplasmosis. Ocul Immunol Inflamm.

[29] Baharivand N, Mahdavifard A, Fouladi RF (2013). Intravitreal clindamycin plus dexamethasone versus classic oral therapy in toxoplasmic retinochoroiditis: A prospective randomized clinical trial. Int Ophthalmol.

[30] Sobrin L, Kump LI, Foster CS (2007). Facs. Intravitreal Clindamycin for Toxoplasmic retinochoroiditis. Retin J Retin Vitr Dis.

[31] Lasave AF, Daz-Llopis M, Muccioli C, Belfort R, Arevalo JF (2010). Intravitreal clindamycin and dexamethasone for zone 1 toxoplasmic retinochoroiditis at twenty-four months. Ophthalmology.

[32] Bor’i A, Mahrous A, Al-Aswad MA, Al-Nashar HY, Nada WM, Wagih M (2018). Intravitreal clindamycin and dexamethasone combined with systemic oral antitoxoplasma therapy versus intravitreal therapy alone in the management of toxoplasma retinochoroiditis: A retrospective study. J Ophthalmol.

[33] Kianersi F, Beni AN, Beni ZN, Ghanbari H (2015). Intravitreal bevacizumab for treatment of choroidal neovascularization secondary to toxoplasmic retinochoroiditis: A case series. Semin Ophthalmol.

[34] Ben Yahia S, Herbort CP, Jenzeri S, Hmidi K, Attia S, Messaoud R (2008). Intravitreal bevacizumab (Avastin) as primary and rescue treatment for choroidal neovascularization secondary to ocular toxoplasmosis. Int Ophthalmol.

[35] Korol AR, Zborovska O, Kustryn T, Dorokhova O, Pasyechnikova N (2017). Intravitreal aflibercept for choroidal neovascularization associated with chorioretinitis: A pilot study. Clin Ophthalmol.

[36] Hegde S, Relhan N, Pathengay A, Bawdekar A, Choudhury H, Jindal A (2015). Coexisting choroidal neovascularization and active retinochoroiditis-an uncommon presentation of ocular toxoplasmosis. J Ophthalmic Inflamm Infect.

[37] Kianersi F, Beni ZN, Beni AN, Heshmatollah G, Ahmadi M (2015). Anti-vascular Endothelial Growth Factor for Choroidal Neovascularization Associated with Toxoplasmosis: A Case Series. Clin Experiment Ophthalmol.

[38] Anaya D, Castro A (2019). Unusual associations with toxoplasmosis: Report of two cases. Pan-American J Ophthalmol.

[39] Santos EMG (2008). Treatment of juxtapapillary toxoplasmic retinochoroiditis with intravitreal clindamycin and dexamethasone injection. Ophthalmology.

[40] Khandwala NS, Hyde RA, Besirli CG (2021). Toxoplasma Retinochoroiditis with Chorioretinal Neovascularization in a Young Patient. Case Rep Ophthalmol.

[41] Wong R, dell’Omo R, Marino M, Hussein B, Okhravi N, Pavesio CE (2009). Toxoplasma gondii: An atypical presentation of toxoplasma as optic disc swelling and hemispherical retinal vein occlusion treated with intravitreal clindamycin. Int Ophthalmol.

[42] Henao-Martínez AF, Franco-Paredes C, Palestine AG, Montoya JG (2018). Symptomatic acute toxoplasmosis in returning travelers. Open Forum Infect Dis.

[43] Martinez CE, Zhang D, Conway MD, Peyman GA (1998). Successful management of ocular toxoplasmosis during pregnancy using combined intraocular clindamycin and dexamethasone with systemic sulfadiazine. Int Ophthalmol.

[44] Fonollosa A, Llorenç V, Artaraz J, Jimenez B, Ruiz-Arruza I, Agirrebengoa K (2016). Safety and efficacy of intravitreal dexamethasone implants in the management of macular edema secondary to infectious uveitis. Retina.

[45] Mushtaq F, Ahmad A, Qambar F, Ahmad A, Zehra N (2019). Primary Acquired Toxoplasma Retinochoroiditis: Choroidal Neovascular Membrane as an Early Complication. Cureus.

[46] Mehta N, Chong J, Tsui E, Duncan JL, Curcio CA, Freund KB (2018). Presumed Foveal Bacillary Layer Detachment in a Patient With Toxoplasmosis Chorioretinitis and Pachychoroid Disease. Retin Cases Brief Rep.

[47] Mathur G, George AE, Sen P (2014). Paediatric choroidal neovascular membrane secondary to toxoplasmosis treated successfully with anti-vascular endothelial growth factor. Oman J Ophthalmol.

[48] Perez AL, Lozada RA, Emanuelli A, Oliver AL (2020). Optical coherence tomography angiography findings in macular toxoplasma retinochoroiditis: A case report. Am J Ophthalmol Case Rep.

[49] Martín García E, Chávarri García JJ, Rodríguez Vicente L, Jiménez del Río B, Guallar Leza SM, del Río Mayor JL (2020). Management of the neovascular choroidal membrane secondary to ocular toxoplasmosis. Arch la Soc Española Oftalmol.

[50] Bawdekar AC, Jindal A, Shah M, Pathengay A (2013). Intravitreal triamcinolone acetonide in management of ocular toxoplasmosis in an HIV patient: A case report. Can J Ophthalmol.

[51] Shah NJ, Shah UN (2011). Intravitreal ranibizumab for the treatment of choroidal neovascularization secondary to ocular toxoplasmosis. Indian J Ophthalmol.

[52] Hosseini SM, Abrishami M, Mehdi Zadeh M (2014). Intravitreal Clindamycin in the Treatment of Unresponsive Zone One Toxoplasmic Chorioretinitis: A Case Report. Iran Red Crescent Med J.

[53] Kim P, Younan N, Coroneo MT (2002). Hypersensitivity reaction to intravitreal clindamycin therapy. Clin Exp Ophthalmol.

[54] Abrishami M, Hosseini MS, Momtahen S, Zamani G (2021). Foveal Regeneration after Treatment of Acute Foveal Toxoplasmic Chorioretinitis. Res Sq.

[55] Dutta Majumder P, Shah A, Madhuravasal Krishnan J, Rishi E, Curi ALL (2021). Driving in Fog without Headlight: Management of a Challenging Case of Presumed Ocular Toxoplasmosis. Ocul Immunol Inflamm.

[56] Ghassemi F, Ghadimi H, Amoli FA, Esfahani MR, Tavakoli V, Fariba G (2014). Diffuse infiltrating retinoblastoma coexisting with ocular toxoplasmosis. Int Ophthalmol.

[57] ishi P, Venkataraman A, Rishi E (2011). Combination photodynamic therapy and bevacizumab for choroidal neovascularization associated with toxoplasmosis. Indian J Ophthalmol.

[58] Theodoropoulou S, Schmoll C, Templeton K, Dhillon B (2012). Atypical toxoplasmic retinochoroiditis. BMJ Case Rep.

[59] Raval V, Rao S, Das T (2018). Anatomical and functional outcomes of pars plana vitrectomy for inflammatory epiretinal membrane surgery in healed toxoplasmosis infection. Indian J Ophthalmol.

[60] Sánchez Vega C, Hernández Obregón D, Reyes Rodríguez M, Hernández F (2014). Agujero macular tras toxoplasmosis retiniana.: Presentación de un caso. Arch la Soc Canar Oftalmol.

[61] Hazan A, Patel RM, Levinson D, Mian U, Gritz DC (2013). A typical bilateral Toxoplasma retinochoroiditis in a bone marrow transplant patient with negative serum titers. J Ophthalmic Inflamm Infect.

[62] Iaccheri B, Fiore T, Papadaki T, Androudi S, Janjua S, Bhaila I (2008). Adverse drug reactions to treatments for ocular toxoplasmosis: A retrospective chart review. Clin Ther.

[63] Ben-Harari RR, Goodwin E, Casoy J (2017). Adverse Event Profile of Pyrimethamine-Based Therapy in Toxoplasmosis: A Systematic Review. Drugs R D.

[64] Handaye-Dessus A, Gattoussi S, Korobelnik JF, Delyfer MN, Rougier MB (2021). Intravitreal clindamycin injection in toxoplasma retinochoroiditis: About 9 cases in the ophthalmology department of the CHU de Bordeaux. J Fr Ophtalmol.

[65] Zhang Y, Lin X, Lu F (2018). Current treatment of ocular toxoplasmosis in immunocompetent patients: A network meta-analysis. Acta Trop.

[66] Fabiani S, Caroselli C, Menchini M, Gabbriellini G, Falcone M, Bruschi F (2022). Ocular toxoplasmosis, an overview focusing on clinical aspects. Acta Trop.

[67] Rajapakse S, Chrishan Shivanthan M, Samaranayake N, Rodrigo C, Deepika Fernando S (2013). Antibiotics for human toxoplasmosis: A systematic review of randomized trials. Pathog Glob Health.

[68] Silveira C, Belfort R, Muccioli C, Holland GN, Victora CG, Horta BL (2002). The effect of long-term intermittent trimethoprim/sulfamethoxazole treatment on recurrences of toxoplasmic retinochoroiditis. Am J Ophthalmol.

[69] Fernandes Felix JP, Cavalcanti Lira RP, Grupenmacher AT, Assis HLG de, Cosimo AB, Nascimento MA (2020). Long-term Results of Trimethoprim-Sulfamethoxazole Versus Placebo to Reduce the Risk of Recurrent Toxoplasma gondii Retinochoroiditis. Am J Ophthalmol.

[70] Fernandes-Cunha GM, Gouvea DR, Fulgêncio G de O, Rezende CMF, da Silva GR, Bretas JM (2015). Development of a method to quantify clindamycin in vitreous humor of rabbits’ eyes by UPLC-MS/MS: Application to a comparative pharmacokinetic study and in vivo ocular biocompatibility evaluation. J Pharm Biomed Anal.

[71] Fernandes-Cunha GM, Rezende CMF, Mussel WN, da Silva GR, Elionai EC, Yoshida MI (2016). Anti-Toxoplasma activity and impact evaluation of lyophilization, hot molding process, and gamma-irradiation techniques on CLH-PLGA intravitreal implants. J Mater Sci Mater Med.

[72] Fernandes-Cunha GM, Fialho SL, da Silva GR, Silva-Cunha A, Zhao M, Behar-Cohen F (2017). Ocular safety of Intravitreal Clindamycin Hydrochloride Released by PLGA Implants. Pharm Res.

[73] Cerqueira Lima GS, Saraiva PGC, Saraiva FP (2015). Current Therapy of Acquired Ocular Toxoplasmosis: A Review. J Ocul Pharmacol Ther.

[74] Ozgonul C, Besirli CG (2016). Recent Developments in the Diagnosis and Treatment of Ocular Toxoplasmosis. Ophthalmic Res.

[75] Pradhan E, Bhandari S, Gilbert RE, Stanford M (2016). Antibiotics versus no treatment for toxoplasma retinochoroiditis. Cochrane Database Syst Rev.

[76] Dunay IR, Gajurel K, Dhakal R, Liesenfeld O, Montoya JG (2018). Treatment of Toxoplasmosis : Historical Perspective , Animal. Clin Microbiol Infect.

[77] Feliciano-Alfonso JE, Muñoz-Ortiz J, Marín-Noriega MA, Vargas-Villanueva A, Triviño-Blanco L, Carvajal-Saiz N (2021). Safety and efficacy of different antibiotic regimens in patients with ocular toxoplasmosis: systematic review and meta-analysis. Syst Rev.

[78] Bonfioli AA, Orefice F (2005). Toxoplasmosis. Semin Ophthalmol.

[79] Bosch-Driessen LH, Verbraak FD, Suttorp-Schulten MSA, Ruyven RLJV, Klok AM, Hoyng CB (2002). A prospective, randomized trial of pyrimethamine and azithromycin vs pyrimethamine and sulfadiazine for the treatment of ocular toxoplasmosis. Am J Ophthalmol.

